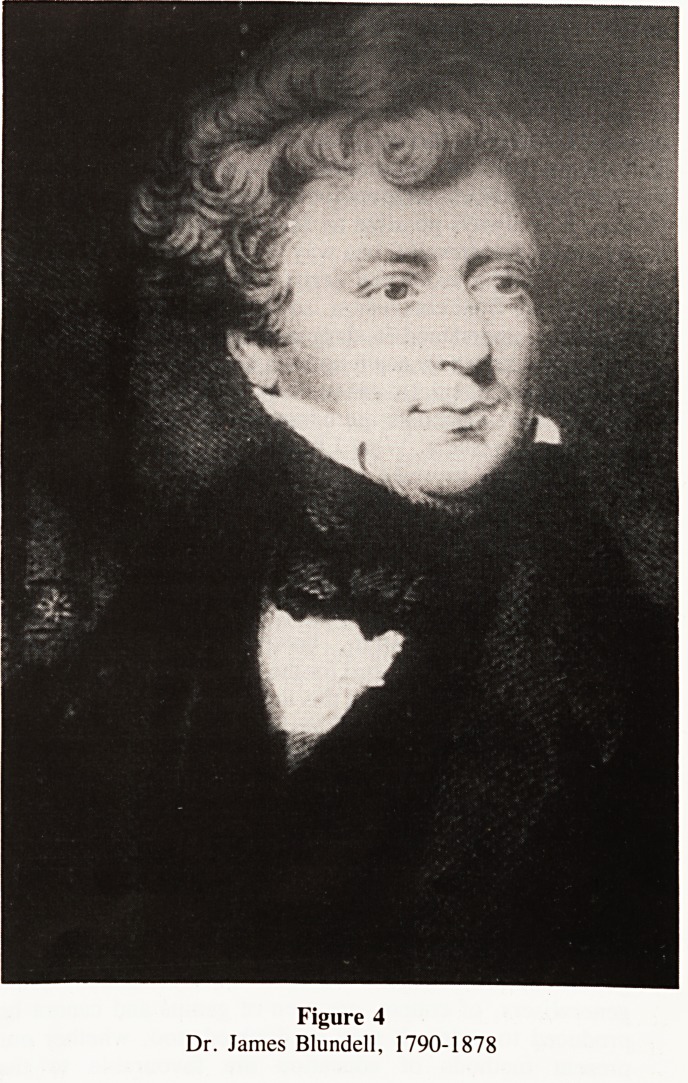# What Is This Life If Full of Care

**Published:** 1992-03

**Authors:** Peter M Dunn

**Affiliations:** Professor of Perinatal Medicine and Child Health University of Bristol


### What is this life, if full of care, we have no time to stand and stare?*

**Peter M, Dunn**
Professor of Perinatal Medicine and Child Health
University of Bristol

William Henry Davies (Fig. 1) was born in May 1871 in Newport at the mouth of the River Usk in South Wales. As a young man he sought work in Bristol and became apprenticed to a picture-frame maker. Becoming restless he crossed the Atlantic in a cattle boat in about 1890 and commenced a life of wandering in the U.S.A., tramping, begging and doing odd-jobs. In fact he became a hobo. This way of life continued until one day, while jumping a goods-train, he fell underneath it and lost a leg. On recovering he returned to England but continued much the same manner of living as a pedlar and street-singer. And that might easily have been his story, the life of a tramp. However during his travels Davies had had time to think, time to observe the beauty of Nature about him and to ponder on the meaning and purpose of life, and he started to write down his thoughts in simple poems, at first on bits of paper and then after his return to England, at an expense that stretched his slender financial resources to their limits, he had them printed in a little book entitled "Nature Poems and Others". These he attempted to sell but no one was interested and in despair he dispatched his whole stock by post to various critics, editors and men of letters. Ninety-nine fell on stony ground. The hundredth arrived on the breakfast table of George Bernard Shaw and was scrutinized by a man with enough talent to recognise that quality in another. He asked Davies to come and see him and after meeting agreed to give him his backing. In fact he wrote the preface to the autobiography which Davies had published the following year. During the remaining years of his life Davies published a number of collections of poems and essays. Eventually he died in 1940 and has his own niche in the Hall of Fame.

I find this story of Davies' inspiring. Here was a man of humble origin and little formal education whose early life was, by most people's standards, misspent. Yet in spite of this he succeeded in achieving distinction and fame as a man of letters. How did this come about? I shall try to answer this question and at the same time take the opportunity of discussing a few of my own interests, namely the importance of leisure, the sterility of much modern education, the need for reflective thinking, and also the desirability of those most human qualities, humanity and humility. Davies possessed both these last qualities and his life of wandering gave him the opportunity to become a reflective philospher, for his mind had not been stunted by the process of schooling which passes today for education. Only last week I noticed in the Centenary memorial display in honour of Beatrix Potter that she had written of herself: "Thankgoodness my education was neglected; I was never sent to school . . . . The reason I am glad I did not go to school is that it would have rubbed off some of my originality".

So let me start by discussing "Leisure" which, indeed, was the name Davies chose for the poem, the first couplet of which I have borrowed as the title for this discussion: "What is this life, if full of care, we have no time to stand and stare?" Most people are so busy that they haven't even time to stop to ask themselves this question with the result that all sense of perspective as to what is, and what is not, important in life has become blurred and ill-defined. Western civilization teaches that to work hard is good and to work so hard that your health breaks down and you die prematurely is virtually to die the death of a martyr. But what do most people mean by work? Something we do each day between 9 and 5 and perhaps also take home with us in the evening and at the weekend. Preferably it should be rather tiresome and exhausting, something that we dislike doing, but still do, bravely, for the sake of our family, our country or even for the good of mankind. To actually enjoy your work is vaguely improper, if not cheating. Indeed some would say that this disqualifies it from being called 'work'. In England I was told several times in the last three years that I was very lucky to be paid a salary because I was doing research work which I enjoyed! To continue, above all you must be seen to be working. It matters very much less whether or not you actually achieve anything. This too I can illustrate from my own experience. During 1964 while collecting data on newborn infants, I was always scurrying backwards and forewards with notebooks, equipment and samples of blood, often late into the night. Many people commended me on how hard I was working. Then, in 1965 I spent nearly 6 months buried in the stack-room beneath the medical library analysing my data, thinking about it and preparing some of it for publication. Long before the six months were up, people were beginning to look at me

**Figure 1**

William Henry Davies, 1871-1940.

**This paper was presented to the Research Fellows of the Cardio- Vascular Research Institute, University of California School of Medicine, San Francisco on December 10th, 1966.*

sideways and ask when I was going to start work again. Yet, as you all know, collecting data is child's play compared to thinking about it. Let me read a quotation from Erasistatus of Alexandria on this subject. He was a very great physician and physiologist who lived some 2300 years ago:

> ''Those who are altogether unaccustomed to research are at the first exercise of their intelligence befogged and blinded and quickly desist owing to fatigue and failure of intellectual power, like those who without training attempt a race. But one who is accustomed to investigation, worming his way through and turning in all directions, does not give up the search, I will not say day or night, but his whole life long. He will not rest but will turn his attention to one thing after another which he considers relevant to the subject under investigation until he arrives at the solution of his problem.''

No discussion of the subject of work can be considered complete without reference to an admirable little book by C. Northcote Parkinson, ersthwhile Professor of History in the University of Malaya. In this book, called ''Parkinson's Law and Other Studies in Administration'', he examines a number a number of facets of the English way of life. I shall just quote the opening paragraph:

> ''Work expands so as to fill the time available for its completion. General recognition of this fact is shown in the proverbial phrase 'It is the busiest man who has time to spare'. Thus, an elderly lady of leisure can spend the entire day in writing and despatching a postcard to her niece at Bognor Regis. An hour will be spent in finding the postcard, another in hunting for spectacles, half-an-hour in a search for the address, an hour and a quarter in composition, and twenty minutes in deciding whether or not to take an umbrella when going to the mailbox in the next street. The total effort that would occupy a busy man for three minutes all told may in this fashion leave another person prostrate after a day of doubt, anxiety and toil.
>
> Granted that work (and especially paperwork) is thus elastic in its demands on time, it is manifest that there need be little or no relationship between the work to be done and the size of the staff to which it may be assigned. A lack of real activity does not, of necessity, result in leisure. A lack of occupation is not necessarily revealed by a manifest idleness. The thing to be done swells in importance and complexity in a direct ratio with the time to be spent.
>
> This fact is widely recognised, but less attention has been paid to its wider implications, more especially in the field of public administration. Politicians and taxpayers have assumed that a rising total in the number of civil servants must reflect a growing volume of work to be done''.

Parkinson then goes on to show that this is not so. For instance, he points out that while the number of ships and sailors in the Royal Navy were halved between 1914 and 1928, during the same period the number of dockyard officials increased by 40%, while the Admiralty officials increased by 78%. Again, in 1935, when the British Empire was at its height, the Colonial Office employed a staff of 372. During the next 30 years the size and number of territories to be administered shrank, until by 1954, outside Africa, there was precious little left. Yet, during this period the staff of Colonial Office officials steadily increased so that by the end it was 500% greater than it had been at the start. He explains this extaordinary phenomenon by stressing that officials want to multiply subordinates, not rivals, and that they make plenty of work for each other so that eventually their original function may become unimportant or even irrelevant. And so we dash around like ants, too busy, maybe, to kiss the wife and children goodbye in the morning and too tired to do so when we get home in the evening; too busy to drive carefully to work, so that some do not even arrive — the ultimate absurdity; far too busy to plan our lives or to consider where we are going.

The medical profession is no better than anyone else; indeed we are probably worse than most and have less excuse in that we ought to know better.

Let me give one or two examples:

Modern medicine concentrates most of its energy and money on the diagnosis and treatment of established disease. So much of our effort, indeed, is spent in this way that we have little time or money left to spend on prevention. Thus, although B.C.G. vaccination has been available for many years there are still in 1966 some 60,000 new tuberculous infections among American children each year. Then in the U.K. some 1,000 children are seen annually at the age of 1 to 3 years with congenital dislocation of the hip. They undergo long, unpleasant, expensive and frequently unsatisfactory treatment when, for a fraction of the cost and suffering they might have been diagnosed at birth and the defect corrected with little effort.

In Great Britain many doctors, especially hospital doctors, tend to look down on colleagues working in the fields of preventive and social medicine. Yet the latter have, with the aid of paramedical help from colleagues in public health departments, done more for the health of the country than any other branch of medicine.

Research workers are often no better, being in such haste to solve problems and to win fame and next year's salary that they frequently fail to plan their experiments properly with the result that they are deemed worthless and need to be repeated. Alternately, the same study may have been done in the past and lie completed and reported in the medical library, had they but found the time to look for It. As Sir Robert Hutchison (Fig. 2) said during his Harveian Oration at the Royal College of Physicians In 1931:

> ''Look round this room in which we are met. It is a noble library indeed but is it not also a mausoleum? And how many facts which men are at present hunting for, and theories which are even now being put forward as new, lie already buried in these shelves''.

**Figure 2.**
Sir Robert Hutchison, 1871-1960

This remark is true to an extent that is hard to credit. I mentioned that I spent some months in the stack-room of the Bristol University Medical Library. Each day I used to take home two or three dust-coverd old tomes and browse through them in the evening by the fire and I never ceased to marvel at the wisdom of the great medical men of the past and the vast amount of knowledge that had been established only then to fall back once more into oblivion. In my own field of the newborn infant I could name at least 20 major so-called discoveries made since I qualified, which were known at the turn of the century or before. For instance, positive pressure respiration through an endotracheal tube was used for resuscitation of the asphyxiated newborn infant more than 200 years ago, while alkalis were administered intravenously for this condition at the beginning of this century. The effects of smoking during pregnancy on the fetus is very much in the news at the moment. However, many of the recent findings on this subject were reported first in the last century.

There are many more classic examples in the literature. In 1666 Mayow described the process of respiration and gave an explanation of its mechanics which might almost have been written today. He showed that only a part of the inhaled air, the igneo-arterial particles, was used in respiration and in burning. More than 100 years later Lavoisier and Priestly were to receive the credit for discovering oxygen. Then Mendel's law of interitance was neglected for more than 40 years. If ever there was a man of genius, it was Mendel. Yet he was unable to pass his exams to become a schoolmaster as he desired and for this reason entered the church which was perhaps fortunate as it gave him time for reflection. Puerperal sepsis provides a third example. The honour of preventing puerperal fever is usually given to Semmelweis of Vienna in the 1840s though truly it should belong to Charles White of Manchester who came nearer the truth and achieved better results than Semmelweis some 80 years earlier.

**Figure 3**
Dr. William Harvey, 1578-1657

It may be argued that all this was due to poor communication in the past. However, there is a great deal of evidence to show that this is not, in fact, likely to be the explanation. Truth has always had to fight its way. Pay heed to William Harvey's reply to Dr. Ent when that worthy gentleman tried to persuade him to publish his second great work 'De Generatione' in 1650:

> "And would you be the man who should recommend me to quit the peaceful haven where I now pass my life, and launch again upon the faithless sea? You know full well what a storm my former lucubrations raised. Much better is it often times to grow wise at home and in private, then, by publishing what you may have amassed with infinite labour, to stir up tempests that may rob you of your sleep and quiet for the rest of your days".

Dr. Harvey (Fig. 3) may be forgiven for this sentiment for, in spite of being the King's physician and for many years a Censor at the Royal College of Physicians, he received more than his share of criticism at the hands of colleagues. In fact, at much the same time as his conversation with Dr. Ent was taking place, Guy Pattin, the French Anatomist, was applying to King Louis XIV to have the circulation theory officially banned in France. In his application he wrote that the theory was "paradoxical, useless, erroneous, impossible, absurd and harmful".

**Figure 4**
Dr. James Blundell, 1790-1878

James Blundell (Fig. 4) of Guy's Hospital, one of the greatest physiologists and experimentors of the last century, said prophetically in respect to blood transfusion which he attempted to introduce in the treatment of severe blood loss in the 1820s: "It will, after undergoing the usual ordeal of neglect, opposition, and ridicule, hereafter be admitted into general practice". Elsewhere Munroe-Smith, describing the introduction of anaesthesia in Bristol in the last century, wrote: ". . . . but these anaesthetics found their way in the practice of Bristol Infirmary very slowly. The surgeons were unwilling to experiment on their patients, and for many years after the use became general (elsewhere), long and painful operations were frequently gone through without (their aid), the patients being carefully strapped down and sometimes large doses of brandy and opium given''. One surgeon is said to have liked to hear a good healthy scream from his patients during an operation, as then he knew that they were still alive! On another subject, antisepsis, Munroe-Smith wrote: ''It is astonishing that, with the results of Lister's treatment of wounds before their eyes, so many eminent surgeons refused for several years to adopt it, and even fought it most vigorously. It is worth noting that the surgeon who most opposed its introduction into the Bristol Infirmary was the last (many years later) to give up the use of the (carbolic acid) spray after he had become a convert''.

Santayana reminds us that 'He who neglects the lessons of history is condemned to repeat them'. You may feel that things have changed; but have they? Let us examine for instance the aircraft industry, one of the least conservative organisations. In retrospect it seems almost incredible that for years the three most imaginative and exciting advances in aircraft design, Whittle's jet engine, Wallis' foldback wings and the hovercraft of Cockerell, were rejected as unsound and impractical by all the foremost authorities.

In medicine we have tended to become a little more subtle in our rejection of new ideas. Doctors who base a great deal of their daily practice on the folklore of the past, shelter behind an impregnable defence of ''There isn't sufficient statistical proof''. Thereby they safeguard themselves against later ridicule and in the meantime maybe even enhance their own reputation as cautious and careful workers. Even when the statistical evidence is provided, as it was in the case of smoking and lung cancer, it is always possible to find a loophole in the design of the study. Incidentally, one way to get a new idea published without attracting scorn and derision is to make your paper, except for the final conclusion, so dull and abstruse that few will read or understand it. Jargon should be used wherever possible and the whole sown up with a statistical analysis that only a pure mathematician can unravel. Judging by the journals, more and more scientists are adopting this method.

But I am digressing. What I have been trying to say is that there is a serious shortage of ''thinkers'' in the world today, people who can dream up new and exciting ideas or who are capable of recognising such ideas when they are placed before them. Let me quote Robert Hutchison again:

> ''Now specialism has been carried to the point of view, and the right hand of science does not know what the left hand is doing. Specialization, however favourable to the accumulation of facts is bad for the philosophy of knowledge. There is in consequence of this too little speculation and too little use of the imagination, and compared with the wealth of knowledge, and the brilliance of observation which it exhibits, most scientific literature is barren of ideas. But most of all we need thinkers. Observation and experiment can give us facts, have indeed given us too many facts. What we require is men with imagination, men of the comtemplative type of Harvey, fertile in hypothesis, who can see the interrelation betweeen the facts and who can bind them into manageable sheaves and induce from them those generalizations which we call natural laws. Great generalisers, of course, are men of genius and cannot be produced to order. It may be doubted, too, whether our present methods of education are favourable to the production of the type of men we most need. To give up the human studies and take to specialism is not the way to train the imagination and tends to result in the production of skilled technicians rather than of educated men''.

Here Hutchison is hinting at what I believe to be the case: that genius is rare not because few men are born with this potential, but because this quality is seldom given the opportunity of developing and bearing fruit. When our children are small and continuously question us, we tend to find it tiresome and discourage them or fob them off with fairy tales. Then, as soon as they are old enough to go to school we commence a process of fact-feeding which continues relentlessly year after year until well into adult life. In Great Britain the compulsary education of an aspiring hospital specialist is not considered complete until he is in his mid-thirties. This acquisition of knowledge, which passes for learning, is enforced by an examination system and an array of selection committees whose primary interest is in the degrees held by the candidate. And in the U.K. some 80 medical degrees are currently available!

The evidence for my belief that genius is not rare is in part historical. Whenever throughout the ages the environment has been temporarily favourable, genius has flourished as in Ancient Greece in the days of Socrates, or Padua in the 16th century. Another remarkable example was provided by France. During the 18th century this country contributed very little to medicine. In 1800 Napoleon disestablished the medical establishment, and overnight France leapt to the fore as the Mecca to which all medical men came. The long list of remarkable men produced in this country during the next 50 years includes Corvisart, Laennec, Dupuytron, Billard, Louis, Bernard and Pasteur to name just a few. Closer to home the success of the Cardiovascular Research Institute here in San Francisco is, above all, attributable to the environment that Dr. Julius Comroe has been created by insisting that we question everything. As a medical historian has written recently: ''It appears that the interest in raising questions at all and then the ability to reliably prove or disprove any offered answers are the determining factors determining progress''. And as James Blundell told his medical students in 1826:

> ''Think for yourselves . . . . do not let my opinion or the opinion of my distinguished colleagues have more weight with you than truth and nature entitle them to. In religion faith is essential; in physiology, a philosophical scepticism . . . . But Gentlemen, it is not enough to think for yourselves and that you ever get together your facts; from these facts principles must be deduced if you are to aspire to the merit of enlarging the sphere of physiological knowledge . . . . Beware of temerity — see what may be done in the dead body — gather facts — form inferences — write little — meditate much''.

''Meditate much'' — almost all the great philosophers have been reflective thinkers who have sought solitude from time to time to pursue their meditations; take, for example, religious philosophers like Buddha and Christ, or scientists like Newton, Darwin and Einstein. Of William Harvey we are told: ''. . . . he was a reflective philosopher. Like Hunter his delight was to think . . . . He would withdraw himself to the leads (attic) of his house in town, or to caves in his garden in the country, in order to indulge in his comtemplation''. Surely in this also he has a lesson for our unreflective time.

In order to meditate it is necessary to have the leisure that William Davies wrote about in his poem. It is not easy to become a philosopher if you are struggling against starvation, though you may become philosophic. However, in many Western countries this situation no longer exists. Machines and computers have taken much of the daily work off our hands. Unfortunately, there is no evidence that there has been any increase in the amount of time spent on reflection. The telephone and the television encroach on our privacy and the weekly day of rest is fast disappearing from our culture. Young people, we are told, are bored and hence the increase in drug addiction, promiscuity and crime. Nor do their elders appear to do much better. If there is an outstanding statesman in world politics today, he is keeping very quiet about his talents. And yet we are faced with gigantic problems like the population explosion that cannot be ignored for much longer; and all the time fingers are hovering over buttons which could annihilate life from this planet.

Last week we were presented by a thought provoking condemnation of the American position in Vietnam. The sad paradox in this unhappy affair was that most Americans supported the war in the name of world peace. While not liking it, they felt that the end justified the means. Although this argument is comforting, we would do well to remember that it was also used to excuse the tortures of the Inquisition and the brutal purges of Communist Russia and the Nazis. What then is the explanation for Vietnam? On domestic issues President Johnson has shown himself to be both liberal and humane. Unfortunately he appears to lack imagination. This would matter less if, before coming to office, he had travelled widely and had met and got to know people of other countries, and especially those of Asia and Africa. Then he would not need imagination. He is no exception in this limitation; the quality of humanity is limited in everyone of us by the scope of our experience or imagination. It's just that the President of the U.S.A. must have much more than most if he is to lead wisely in foreign affairs. Let me illustrate this: suppose the Viet Cong had infiltrated into California in the way they have in South Vietnam. It is unthinkable that the American armed forces would still use the same methods to destroy them that they are at present using abroad. Imagine the reaction in the rest of America to the use of napalm and phosphorus bombs in California. The point is that they could imagine what it would be like because California comes within their experience. This is why travel abroad as well as a universal language are very high on my scale of priorities for achieving world peace.

I want now to dwell for a moment on the word humanity, for the possession of this quality and its counterpart, inhumanity, distinguish the human race from other forms of life. To my mind the pursuit of humanity should be the principle purpose in life. This thought is not new. It has, of course, been the message of practically every great religious leader since Isaiah and Buddha. It offers, I think, the only hope of solving the problems that confront us. As doctors we have an especial obligation to set an example to the rest of mankind. I wonder if we always do so. Let me remind you of Webster's definition of 'humanity': "a disposition to relieve distress and to treat all creatures with kindliness". Albert Schweitzer called it 'reverence for life' and it was at the heart of everything he tried to do at Lamberaine in the Congo. His deliberate neglect of material values, which so frustrated the doctors who thought only of saving life was, I think, his way of underlining a much greater need which is man's need to understand the meaning and purpose of life around him. It is something that medical men and scientists tend to forget. Let me quote, for instance, from a recent fair and well-balanced article by Catherine Roberts on the use of animals in medical research:

> "One of the basic concepts of the scientific outlook has always been that the end justifies the means, and now in the twentieth century this concept has finally transcended the realm of science to become an intregral part of the world outlook. Most people today, be they members of the scientific profession or not, would hold that the desire to restrict or hamper the progress of medical research must stem from a profound lack of knowledge, insight, or humanism, for a pursuit which endeavours to improve the health of man can only be noble, idealistic and worthy of unlimited support. In other words, since science **can** extend its knowledge in order to alleviate more human pain and prolong human lives, it **must** do so — and as rapidly as possible and by all means at its disposal. I disagree. I believe that our Scientific Age needs not so much to extend its intellectual frontiers as to become aware of man's ultimate goal: to become more human. We can choose between increased factual knowledge gained by wholly unrestricted scientific progress, together with all the benefits to mankind thereby accruing, or increased humanness. But we cannot have both, for the two are incompatible. With regard to the specific problem of animal experimentation, consider what is being written today in Sweden: 'To reap for one's own benefit advantages through means which involve suffering and fear in defenceless living creatures must be felt to be a heavy burden of guilt for any humanely disposed person. No one can neglect the question, "With what right do we do this, we, who call ourselves human beings?" Herewith the problem is extended from the purely scientific plane to the ethical and the universal and becomes a problem which every man not only has the right but the duty to consider".

There is no time left to discuss the importance of humility but I thought I would end by reading the epitaph on the tomb of the Reverend Thomas Malthus which, I think, illustrates the value of this quality. Malthus was born exactly 200 years ago. He was a shy and humble man with a hare lip and cleft palate. In 1798 he published his "Essay on the Principle of Population' in which he foretold with uncanny prescience the difficulties which we face but do not face up to today. In his own lifetime his views were for the most part treated with scorn. This epitaph lies in Bath Abbey only a few miles away from my home in Bristol. It was written by his great friend, Bishop Otter, and speaks for itself:

> "Sacred to the memory of the Rev. Thomas Malthus
> Long known to the lettered world by his admirable writings on the social branches of political economy particularly by his essay on population.
>
> One of the best men and truest philosophers of any age or country:
> Raised by native dignity of mind;
> Above the misrepresentations of the ignorant and the neglect of the great,
> He lived a serene and happy life, devoted to the pursuit and communication of the truth; supported by a calm but firm conviction of the usefulness of his labours; content with approbation of the wise and good.
>
> His writings will be a lasting monument of the extent and correctness of his understanding.
>
> The spotless integrity of his principles, the equity and candour of his nature, his sweetness of temper, urbanity of manners and tenderness of heart, his benevolence and his piety are the still dearer recollections of his family and friends".

## Figures and Tables

**Figure 1 f1:**
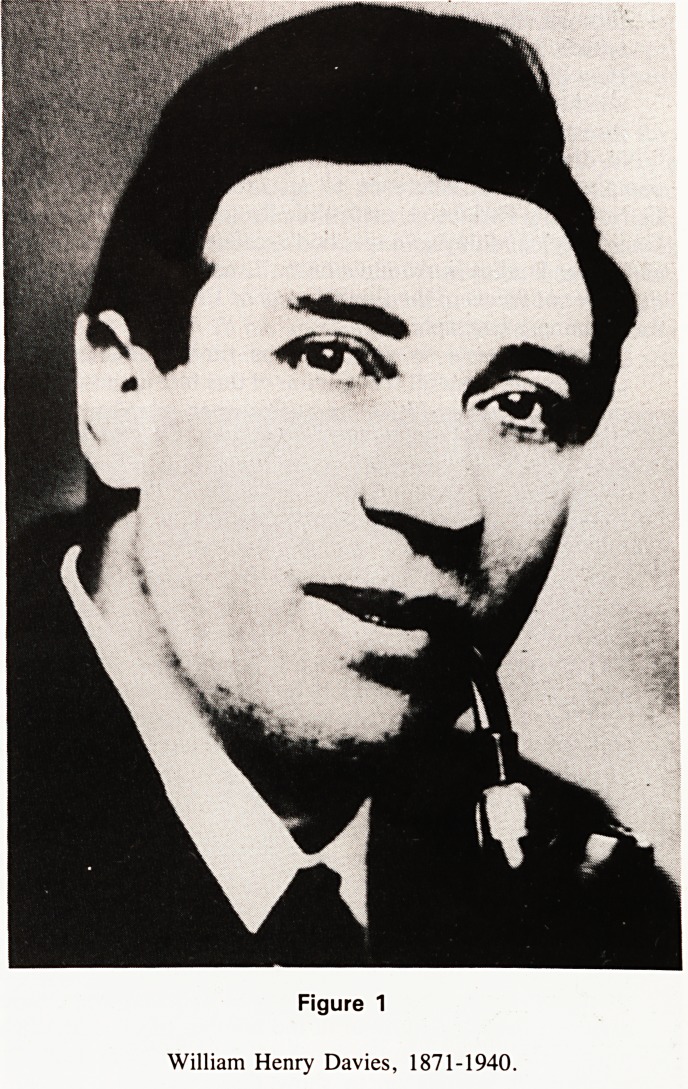


**Figure 2. f2:**
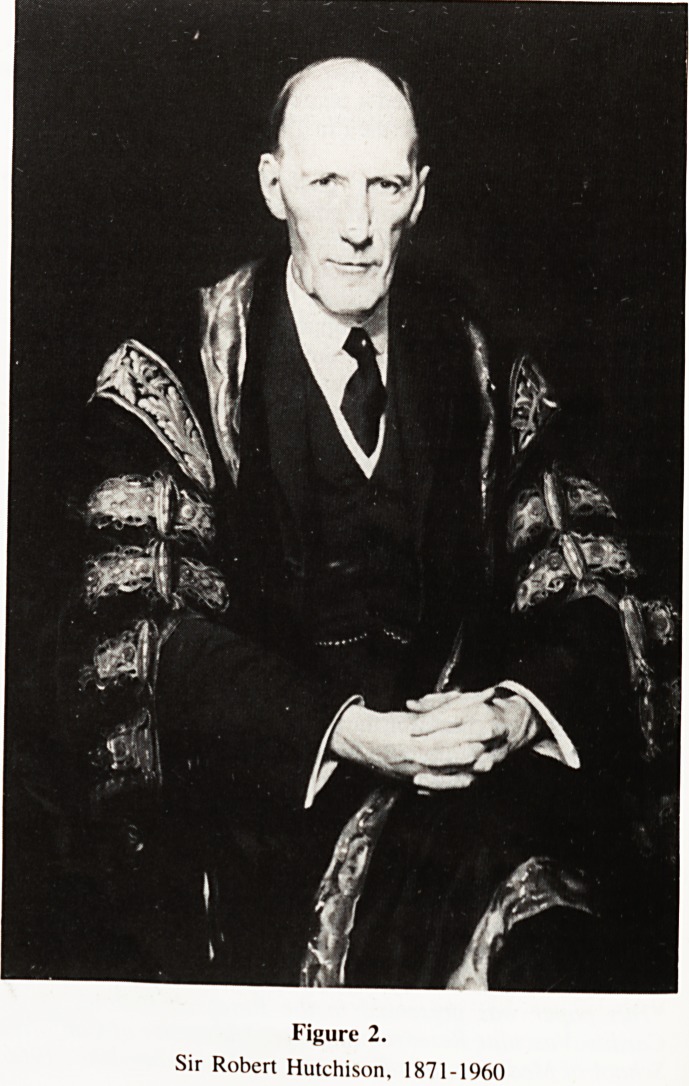


**Figure 3 f3:**
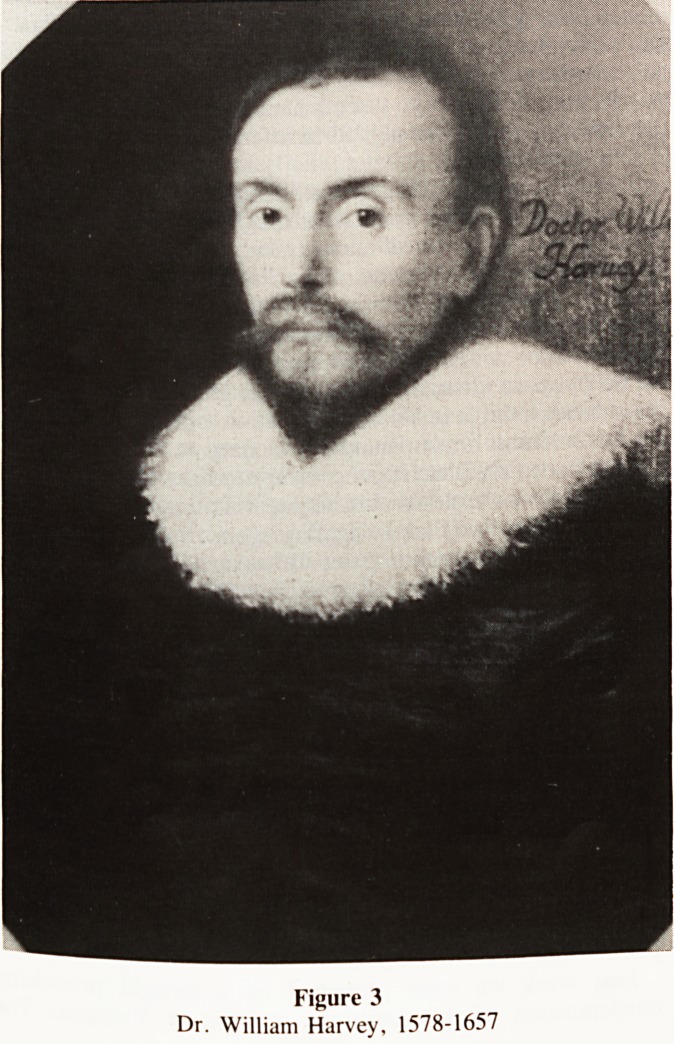


**Figure 4 f4:**